# COVID-19 pandemic response in South American countries: lessons learned and recommendations for health strengthening

**DOI:** 10.17843/rpmesp.2026.432.15674

**Published:** 2026-06-25

**Authors:** Víctor Suárez-Moreno

**Affiliations:** 1 Universidad Nacional Mayor de San Marcos, Lima, Peru.

**Keywords:** COVID-19, Pandemics, Pandemic Preparedness, Health Systems, South America, Learning Health System

## Abstract

The COVID-19 pandemic represented the greatest health and public management challenge of the 21st century for health systems in South America. Peru was one of the countries with the highest mortality rates globally during 2020 and 2021, a situation that evidenced deep structural gaps in its health system. This special article synthesizes the available scientific evidence on the response and lessons learned in South American countries facing the pandemic, based on a literature review published between 2020 and February 2025. It analyzes non-pharmacological interventions, the functioning of health services, the fight against the infodemic, social support, the use of telehealth, vaccination campaigns, and the innovations applied. Responses varied significantly among countries in terms of timeliness, scope, and effectiveness, allowing for the identification of key areas of intervention and replicable good practices. Among the most relevant lessons, the highlights include the need to strengthen critical health infrastructure, institutionalize telehealth, actively combat the infodemic, and promote research and innovation as strategic components of the response.

## INTRODUCTION

On January 30, 2020, the World Health Organization (WHO) declared COVID-19 a public health emergency of international concern ^1^. On March 11 of the same year, it declared it a pandemic ^2^. In immediate response, on March 15, the Peruvian government issued Supreme Decree No. 044-2020-PCM, declaring a National State of Emergency ^3^. The COVID-19 pandemic tested the response capacity of Peru and other countries, not only in the field of health but in all spheres of public management, as well as international coordination mechanisms for a joint response.

Globally, more than seven million deaths attributed to COVID-19 were reported, of which more than 200,000 occurred in Peru ^4^. During the years 2020 and 2021 (the worst years of the pandemic due to the limited availability of vaccines), Peru was among the five countries with the highest COVID-19-associated mortality rate in the world ^5^. This situation reflected previous structural deficiencies of the health system that the health crisis dramatically exacerbated.

It is highly probable that a new pandemic may occur in the not too distant future. Recently, sporadic human cases of highly pathogenic avian influenza (HPAI) A(H5N1) virus have been reported, which are concerning due to their rapid spread among different bird and mammal species, as well as their global expansion, although cases of sustained human-to-human transmission have not yet been confirmed ^6^. Given this scenario, countries should be taking actions to improve their response capacity, based on the experience of the recent pandemic.

This article aims to synthesize the published evidence on the response and lessons learned from the COVID-19 pandemic in South American countries, with the purpose of contributing to the analysis and preparation of plans for future pandemics, with emphasis on the implications for Peru.

## METHODOLOGICAL APPROACH

A rapid review of the scientific literature describing the response of South American health systems to the COVID-19 pandemic was conducted. The search was performed in MEDLINE, prioritizing systematic and narrative literature reviews, as well as original studies published between January 2020 and February 2025. After selection based on thematic relevance and methodological quality criteria, 13 publications were included. The topics addressed included: non-pharmacological interventions, health service response, telehealth, social support, infodemic, vaccination, and innovation. The detailed characteristics of the selected articles are presented in the supplementary material. It is recognized that this rapid review may present selection biases; to minimize them, the use of literature reviews as the main source of evidence was prioritized.

## RESULTS

### Non-pharmacological interventions

The first measures adopted in the countries of the region involved social distancing and the use of masks. Bolivia, Ecuador, and Peru were among the countries that most promptly implemented these measures ^7^^,^^8^, while Brazil showed a low level of coordination between the federal government and the states ^9^. As the pandemic was declared, countries were implementing quarantine. Brazil and Mexico were the last to establish it; Brazil showed the greatest subnational variation. Regarding reopening, Brazil was the first, while Peru waited until October 2020 and Argentina was the last to reopen (November 2020) ^7^.

Economic measures to support families were also common in countries, although insufficient. In the case of Peru, with more than 70% of economic activity concentrated in the informal sector, non-compliance with isolation was more evident, with numerous people breaking quarantine to move to their provinces of origin, compromising the effectiveness of the restrictions.

A systematic review indicates that the non-pharmacological interventions most consistent in achieving results in the control of the pandemic were social distancing, confinement, and the use of masks ^10^.

### Response of health services

All countries presented a gap between the availability of ICU beds and ventilators, and the demand generated by the pandemic. Peru was the country with the lowest availability of these resources (table 1) ^9^. However, it was among those that achieved the greatest increase: 349% in ICU beds and 83% in ventilators in the first 100 days of the pandemic ^9^. Oxygen supply was another critical problem. Brazil needed 340,000 oxygen cylinders per day; Argentina, Colombia, Mexico, and Peru required approximately 100,000 cylinders each, with families frequently bearing the costs ^11^.

**Table 1:** Health system resources of five selected countries at the start of the pandemic.

Indicator	Brazil	Chile	Colombia	Ecuador	Peru
Physicians per 100,000 inhabitants.	1.8	2.5	2.2	2.0	1.3
Nurses per 1,000 inhabitants.	1.5	2.7	1.3	2.5	2.4
Beds per 1,000 inhabitants.	2.3	2.1	1.7	1.5	1.6
ICU beds per 100,000 inhabitants.	17.0	5.2	10.8	6.8	2.5
Ventilators per 100,000 inhabitants.	29.6	6.8	10.8	10.5	0.9

Adapted from Benítez *et al.*^(^^9^^)^.

UCI: Intensive Care Unit.

Regarding diagnostic tests, Chile and Peru registered the highest number of daily tests per population between April and May 2020. Both countries initially had only one molecular biology laboratory; however, by August 2020, the number of enabled laboratories increased to 112 in Chile and 25 in Peru ^9^.

At the beginning of the pandemic, the use of drugs with a supposed effect against COVID-19 became widespread, such as ivermectin, chloroquine, and azithromycin, based on scarce evidence. Although solid evidence against their use became available later, this practice remained widespread ^11^. In Peru, 52.2% of people with COVID-19 symptoms did not attend any health service during the first year of the pandemic. Among the main factors associated with this behavior, residence in urban or rural jungle areas was identified, while factors such as being over 50 years old, having secondary or higher education, and having health insurance behaved as protective factors ^12^.

Regarding out-of-pocket expenditure and catastrophic health expenditure, a comparative study between Mexico and Peru showed that the proportion of households with catastrophic expenditure increased in both countries during the pandemic (Mexico: 3.3% to 5.6%; Peru: 8.7% to 9.6%), with consistently higher levels in Peru ^13^^,^^14^. In the latter, the main factors associated with higher out-of-pocket expenditure were the type of insurance (particularly the Seguro Integral de Salud), the presence of illnesses in the household, a positive result for COVID-19, and the presence of older adults in the household ^14^.

The elements that best worked to improve the response of the health system in the region included: human resource training, public-private coordination, robust epidemiological surveillance systems, rapid construction of field hospitals, reconversion of hospital beds, scaling up of laboratory capacities, and science-based national coordination mechanisms ^15^.

### Telehealth

The pandemic caused a significant reduction in the use of health services, especially primary care, as a result of the quarantine and the closure of establishments. To compensate for this deficit, countries promoted or strengthened their telemedicine services; in this context, Peru and Chile stood out for the greater development of this strategy ^9^. The technologies used were diverse, including phone calls, videoconferences, mobile applications, emails, text messages, web pages, and teleradiology software. These tools were used for purposes of prevention, screening, triage, diagnosis, treatment, and follow-up. However, its implementation faced multiple barriers, mainly related to connectivity, digital literacy, and regulatory frameworks ^16^.

### Social Support Response

The pandemic unfolded in a context of high social and economic inequality. Latin America is one of the most unequal regions in the world, where highly vulnerable groups coexist: undocumented migrants, rural and indigenous populations, Afro-descendants, and low-income households. A group of experts recommended improving social and economic protection for these groups in a targeted and culturally relevant manner ^15^. In particular, among Venezuelan migrants, some of the lowest vaccination coverages in the region were recorded, which evidences persistent gaps in access to health services.

### Infodemic

The massive circulation of false and unverified information generated uncertainty in the population regarding prevention measures, therapeutic management, and the real situation of the pandemic. Most countries implemented strategies to combat the infodemic, though with different scopes. Argentina developed the web platform “Confiar”, which included sections to verify statements, identify fake news in circulation, and provide guidance on how to detect misinformation. For its part, Brazil enabled a WhatsApp number for inquiries answered by technical teams ^17^.

In contrast, Peru was one of the few countries that did not adopt explicit measures to address the infodemic, limiting itself to announcing criminal sanctions of two to four years of imprisonment for the dissemination of false news related to the pandemic ^18^. Despite this, an evaluation of the infodemic risk index positioned Peru with the highest risk level in the region (99.1%) ^10^. An analysis of the causes and countermeasures to face this problem proposes a broad framework that includes health literacy, coordinated work with mass media platforms, risk communication through accredited spokespersons, and clear guidance to the population ^19^.

### Vaccination

COVID-19 vaccines were slow to arrive in Latin America and the Caribbean. During the first half of 2021, vaccination coverage in the region was significantly lower than in Europe and North America, mainly due to the limited availability of doses. However, in later stages, the region achieved one of the highest complete vaccination coverages (two doses) worldwide ^11^. In Peru, with the aim of expanding vaccine access, the LEGADO Project implemented vaccination centers at various points, achieving user satisfaction levels exceeding 90% ^20^.

### Innovation and research

In Peru, the National Council for Science, Technology and Innovation (CONCYTEC) called for competitive research funds aimed at pandemic control, reaching an investment of USD 2.9 million. The main topics funded included technological innovation, telemedicine and mobile health, as well as epidemiological and social studies. However, the impact of these projects on pandemic control, nor on preparedness for future health emergencies, has not been formally evaluated ^21^.

One of the most notable innovations was the design of the COVOX oxygen concentrator, jointly developed by the Pontificia Universidad Catolica del Peru (PUCP) in collaboration with the DIACSA company. This device releases oxygen at a flow rate of 15 L/min (±1.5), superior to conventional high-flow concentrators (10 L/min), which contributed to mitigating oxygen scarcity during critical moments of the pandemic ^22^. In contrast, countries such as India managed strategic funds for research and innovation, promoted government-academia-industry alliances, and achieved significant contributions in diagnostic kits, vaccines, and medicines. Furthermore, their researchers generated around 8% of systematic reviews and meta-analyses globally during the first year of the pandemic ^23^.

### Mortality from COVID-19

**Figure 1:**
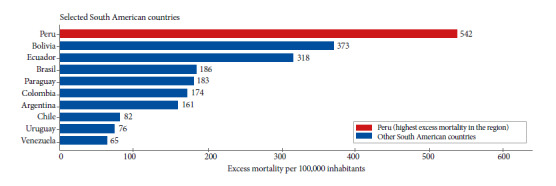
Excess mortality per 100,000 inhabitants, 2020-2021 in selected South American countries.

Source: World Health Organization. Global excess deaths associated with COVID-19 ^5^.

According to WHO data ^4^, Peru was the South American country with the highest excess mortality per 100,000 inhabitants during 2020 and 2021, followed by Bolivia and Ecuador (Figure 1). This situation was directly related to the lower initial availability of critical resources (especially mechanical ventilators and ICU beds) compared to other countries in the region.

## DISCUSSION

The responses of South American countries to the COVID-19 pandemic varied in timeliness, scope, and impact, with disparate mortality outcomes reflecting previous structural differences in their healthcare systems. A determining factor was the level of preparedness of the healthcare system before the onset of the pandemic. Peru, which had the lowest rate of mechanical ventilators per inhabitant in the region, recorded the highest mortality; in contrast, countries better equipped with critical resources showed better outcomes. It has been identified that the main resources that define pandemic preparedness include the availability of hospital and intensive care beds, mechanical ventilation, human resources in health, medications, vaccines, diagnostic capacity, and contact tracing ^21^.

Non-pharmacological interventions, particularly social distancing, lockdown, and mask use, were the most consistent measures in controlling transmission according to available evidence ^22^. However, their effectiveness was limited by socioeconomic factors, such as high labor informality in Peru, which compromised compliance with the lockdown.

The pandemic also evidenced the potential of telehealth as a strategic component of the health response, especially in the care of chronic diseases and case follow-up. The use of these technologies during the pandemic was broad and varied, and there are multiple barriers to overcome for its sustained extension, including connectivity, regulation, and digital literacy ^24^.

The infodemic emerged as a phenomenon parallel to the pandemic, with direct consequences on population behavior and the use of medicines without evidence. The Peruvian case is paradigmatic: being the country with the highest risk of infodemic in the region, it lacked explicit strategies to combat it. This contrasts with the experience of Argentina and Brazil, which developed active verified communication tools ^16^. In this context, public health information management must be integrated as an essential component in pandemic response plans.

Regarding innovation and research, substantive differences were observed between countries. While India integrated research as a pillar of its response, the countries of the region, including Peru, had limited contributions. The experience of the COVOX concentrator demonstrates the potential of local innovation, but its scaling required greater articulation between academia, industry, and government ^20^^,^^23^.

Finally, leadership and governance were key factors in driving the response. Asian countries, with previous experience in epidemics, such as SARS in 2003, showed greater effectiveness, particularly those with efficient public administration and with technical pandemic management committees ^25^. The complexity of the health crisis makes it pertinent to rethink vertical leadership models towards more horizontal, interdisciplinary, and intersectoral schemes.

## LESSONS LEARNED

The experience of the COVID-19 pandemic in South America provides a set of lessons that should guide preparation for future large-scale health events:

1. Critical health infrastructure (ICU beds, ventilators, oxygen supply, and diagnostic laboratories) constitutes a necessary condition to respond effectively to a pandemic. The pre-existing structural gap in Peru was a determining factor in its high mortality rate. Investment in this infrastructure must not be reactive but sustained.

2. Non-pharmacological interventions, especially lockdown and social distancing, are effective in controlling transmission, but their effectiveness is conditioned by socioeconomic factors such as labor informality. Health policies must be articulated with economic and social protection policies.

3. Telehealth proved to be a valuable tool to ensure continuity of care during the pandemic. Its institutionalization as a permanent component of the health system (and not as an emergency response) requires regulatory frameworks, investment in connectivity, and staff training.

4. The infodemic is a phenomenon as dangerous as the epidemic itself. The absence of active risk communication and misinformation management strategies in Peru contributed to the massive use of medications without evidence and to non-compliance with preventive measures. Public health communication must be integrated as a transversal axis of the response.

5. Vaccination, despite delays in access to doses, was the intervention with the greatest impact on reducing mortality. Equity in vaccine access (including vulnerable populations such as migrants and indigenous communities) must be explicitly incorporated into immunization plans.

6. Local research and innovation, when articulated with the needs of the response, can generate high-impact solutions. However, these initiatives require planning, sustained financing, and mechanisms for translating evidence into public policy.

7. Technical and political leadership, intersectoral coordination, and evidence-based governance were decisive in the top-performing countries. The pandemic demonstrated the need to establish and strengthen permanent technical committees for health risk management.

## RECOMMENDATIONS

Based on the lessons learned, the following recommendations are formulated to strengthen the preparedness of Peru and the countries in the region against future pandemics:

1. Closing the critical health infrastructure gap. Peru must establish concrete and funded targets to increase its ratios of ICU beds, mechanical ventilators, and laboratory capacity, using OECD standards and regional comparative experiences as references.

2. Institutionalizing telehealth as a permanent component of the system. This implies developing updated regulatory frameworks, ensuring the interoperability of information systems, investing in connectivity infrastructure in rural areas, and training health personnel.

3. Developing a national risk communication and infodemic strategy. The country must have an official information verification platform, accredited technical spokespersons, and health literacy programs for the general population and vulnerable groups.

4. Strengthening social protection for vulnerable groups. Pandemic response plans must include specific economic and social protection components for informal workers, migrants, indigenous communities, and older adults.

5. Creating a permanent fund for public health research and innovation. Peru must have stable funding for research oriented toward preparedness and response to health emergencies, promoting alliances among government, academia, and industry.

6. Establishing a Permanent National Technical Committee for Epidemic and Pandemic Preparedness, with a clear technical mandate, independence from the political cycle, and the capacity for immediate activation in emergency contexts. This committee should periodically update the National Pandemic Response Plan.

7. Promoting equity as a guiding principle of the health response. Public health interventions (diagnosis, treatment, vaccination, social support) must be designed with an equity approach, guaranteeing access for all populations, especially the most vulnerable.

## CONCLUSIONS

The COVID-19 pandemic evidenced the structural gaps in health systems in South America, with Peru being the most affected country in terms of mortality. The heterogeneity of responses among countries allowed identifying protective factors and replicable practices such as greater availability of critical resources, technical leadership articulated with evidence, timely non-pharmacological interventions, active strategies to combat infodemia, accelerated adoption of telehealth, and investment in local research and innovation.

Five years after the start of the pandemic, Peru faces the imperative to transform the lessons learned into concrete public policies. Preparedness for future pandemics cannot be based solely on reactive responses; it requires sustained investment in infrastructure, human resources, surveillance systems, risk communication, and social protection. The scenario of a new pandemic of avian influenza or another emerging agent is not unlikely, and the cost of inaction has already been demonstrated in human lives.

The pandemic also demonstrated that, although it is possible to reduce transmission and develop vaccines with unprecedented speed globally, this does not prevent the demand for critical services from exceeding the response capacity of weak health systems. Therefore, the strengthening of the Peruvian health system must be a State priority, built on acquired experience, scientific evidence, and commitment to health equity.
