# Supplementary material for: COVID-19 pandemic response in South American countries: lessons learned and recommendations for health strengthening
Source: Rev Peru Med Exp Salud Publica. 2026 Jun 25;43(2):260–6. doi: 10.17843/rpmesp.2026.432.15674 (PMC13456919; doi:10.17843/rpmesp.2026.432.15674)
Supplement: Supplementary file 1 [file rpmesp-43-02-15674-s001-es.docx]

**MATERIAL SUPLEMENTARIO**

**Tabla S1. Características de los artículos seleccionados en la revisión de literatura**

| **Autor (referencia)** | **Año** | **Países** | **Objetivo** | **Intervenciones descritas** |
| --- | --- | --- | --- | --- |
| Alvarez-Risco A, et al (16) | 2020 | Perú | Describir noticias falsas de COVID-19 y la respuesta del gobierno. | Infodemia. |
| Benítez MA, et al (9) | 2020 | Brasil, Chile, Colombia, Ecuador, Perú | Analizar contexto prepandemia, respuesta gubernamental y resultados sanitarios. | Intervenciones no farmacológicas, servicios de salud, apoyo social. |
| Garcia PJ, et al (8) | 2020 | Argentina, Brasil, Chile, Colombia, Costa Rica, Ecuador, México, Perú | Discutir contexto y respuesta inicial en ocho países de América Latina. | Intervenciones no farmacológicas, servicios de salud, apoyo social. |
| Haraki CAC (15) | 2021 | 10 países de Sudamérica | Identificar estrategias de los Ministerios de Salud para gestión de la infodemia. | Infodemia. |
| Hernández-Vásquez A, et al (11) | 2021 | Perú | Estimar prevalencia del no uso de servicios de salud con síntomas de COVID-19. | Respuesta de los servicios de salud. |
| Moya-Salazar J, et al (18) | 2021 | Perú | Analizar priorización de proyectos de investigación ante la pandemia. | Innovación. |
| Knaul FM, et al (7) | 2022 | Argentina, Bolivia, Brasil, Chile, Colombia, Ecuador, México, Perú | Comparar intervenciones no farmacéuticas dentro y entre países. | Intervenciones no farmacológicas. |
| Schwalb A, et al (10) | 2022 | Latinoamérica y Caribe | Explorar desempeño de la región durante los dos primeros años de la pandemia. | Servicios de salud, apoyo social, infodemia, vacunación. |
| Espinosa A, et al (17) | 2023 | Perú | Analizar satisfacción con el modelo de vacunación de LEGADO. | Vacunación. |
| Haakenstad A, et al (12) | 2023 | Bielorrusia, México, Perú, Rusia, Vietnam | Analizar gasto catastrófico en salud durante la pandemia. | Apoyo social. |
| Quispe Mamani JC, et al (13) | 2023 | Perú | Establecer determinantes del gasto de bolsillo en hogares peruanos. | Apoyo social. |
| Ramírez Varela A, et al (14) | 2023 | Argentina, Brasil, Colombia, Perú | Explorar preparación y respuesta pandémica según contexto político-sanitario. | Servicios de salud, apoyo social, innovación. |
| Rubio J, et al (19) | 2023 | Perú | Explicar principio y validación del concentrador de oxígeno COVOX. | Innovación. |
