# Supplementary material for: COVID-19 pandemic response in South American countries: lessons learned and recommendations for health strengthening
Source: Rev Peru Med Exp Salud Publica. 2026 Jun 25;43(2):260–6. doi: 10.17843/rpmesp.2026.432.15674 (PMC13456919; doi:10.17843/rpmesp.2026.432.15674)
Supplement: Translation

# Translation: Respuesta frente a la pandemia de COVID-19 en los países de Sudamérica: lecciones aprendidas y recomendaciones para el fortalecimiento sanitario

**Authors:** Víctor Suárez-Moreno

**Affiliations:** 1 Universidad Nacional Mayor de San Marcos, Lima, Perú.

**Keywords:** COVID-19, Pandemias, Preparación para una Pandemia, Sistemas de Salud, América del Sur, Aprendizaje del Sistema de Salud

## Abstract

La pandemia de COVID-19 representó el mayor reto sanitario del siglo XXI para los sistemas de salud y la gestión pública en Sudamérica. Perú fue uno de los países con mayor tasa de mortalidad a nivel global durante 2020 y 2021, situación que evidenció profundas brechas estructurales en su sistema de salud. Este artículo especial sintetiza la evidencia científica disponible sobre la respuesta y las lecciones aprendidas en los países sudamericanos ante la pandemia, a partir de una revisión de literatura publicada entre 2020 y febrero de 2025. Se analizan las intervenciones no farmacológicas, el funcionamiento de los servicios de salud, el combate a la infodemia, el apoyo social, el uso de telesalud, las campañas de vacunación y las innovaciones aplicadas. Las respuestas variaron significativamente entre países en términos de oportunidad, alcance y efectividad, lo que permitió identificar áreas clave de intervención y buenas prácticas replicables. Entre las lecciones más relevantes destacan la necesidad de fortalecer la infraestructura crítica de salud, institucionalizar la telesalud, combatir activamente la infodemia y promover la investigación e innovación como componentes estratégicos de la respuesta.

## INTRODUCCIÓN

El 30 de enero de 2020, la Organización Mundial de la Salud (OMS) declaró al COVID-19 como una emergencia de salud pública de importancia internacional ^1^. El 11 de marzo del mismo año lo calificó como pandemia ^2^. En respuesta inmediata, el 15 de marzo, el gobierno peruano emitió el Decreto Supremo N.° 044-2020-PCM, declarando el Estado de Emergencia Nacional ^3^. La pandemia de COVID-19 puso a prueba la capacidad de respuesta del Perú y de los demás países, no solo en el campo de la salud sino en todas las esferas de la gestión pública, así como los mecanismos de coordinación internacional para una respuesta conjunta.

A nivel global se reportaron más de siete millones de muertes atribuidas al COVID-19, de las cuales más de 200 000 ocurrieron en el Perú ^4^. Durante los años 2020 y 2021 (los peores años de la pandemia por la limitada disponibilidad de vacunas) Perú estuvo entre los cinco países con la tasa de mortalidad asociada a COVID-19 más alta del mundo ^5^. Esta situación reflejó deficiencias estructurales previas del sistema de salud que la crisis sanitaria exacerbó de manera dramática.

Es altamente probable que pueda ocurrir una nueva pandemia en un futuro no muy lejano. Recientemente se han reportado casos humanos esporádicos de virus influenza aviar altamente patogénico (HPAI) A(H5N1), que preocupan por su rápida extensión entre diferentes especies de aves y mamíferos, así como su expansión global, aunque aún no se han confirmado casos de transmisión sostenida de humano a humano ^6^. Frente a este escenario, los países deberían estar tomando acciones para mejorar su capacidad de respuesta, basándose en la experiencia de la pandemia reciente.

Este artículo tiene como objetivo sintetizar la evidencia publicada sobre la respuesta y las lecciones aprendidas de la pandemia de COVID-19 en los países de Sudamérica, con la finalidad de contribuir al análisis y a la preparación de planes para futuras pandemias, con énfasis en las implicaciones para el Perú.

## ABORDAJE METODOLÓGICO

Se realizó una revisión rápida de la literatura científica que describiera la respuesta de los sistemas de salud de Sudamérica frente a la pandemia de COVID-19. La búsqueda se efectuó en MEDLINE, priorizando revisiones de literatura sistemáticas y narrativas, así como estudios originales publicados entre enero de 2020 y febrero de 2025. Tras la selección por criterios de relevancia temática y calidad metodológica, se incluyeron 13 publicaciones. Los temas abordados incluyeron: intervenciones no farmacológicas, respuesta de los servicios de salud, telesalud, apoyo social, infodemia, vacunación e innovación. Las características detalladas de los artículos seleccionados se presentan en el material suplementario. Se reconoce que esta revisión rápida puede presentar sesgos de selección; para minimizarlos se priorizó el uso de revisiones de literatura como fuente principal de evidencia.

## RESULTADOS

### Intervenciones no farmacológicas

Las primeras medidas adoptadas en los países de la región tuvieron que ver con el distanciamiento social y el uso de mascarillas. Bolivia, Ecuador y Perú estuvieron entre los países que más tempranamente implementaron estas medidas ^7^^,^^8^, en tanto que Brasil presentó un bajo nivel de coordinación entre el gobierno federal y los estados ^9^. A medida que se declaró la pandemia, los países fueron implementando la cuarentena. Brasil y México fueron los últimos en establecerla; Brasil mostró la mayor variación subnacional. Respecto a la reapertura, Brasil fue el primero, mientras que Perú esperó hasta octubre de 2020 y Argentina fue el último en reabrir (noviembre de 2020) ^7^.

Las medidas económicas para apoyar a las familias también fueron comunes en los países, aunque insuficientes. En el caso de Perú, con más del 70% de la actividad económica concentrada en el sector informal, fue más evidente el incumplimiento del aislamiento, con numerosas personas que rompieron la cuarentena para movilizarse a sus provincias de origen, comprometiendo la efectividad de las restricciones.

Una revisión sistemática señala que las intervenciones no farmacológicas con mayor consistencia en lograr resultados en el control de la pandemia fueron el distanciamiento social, el confinamiento y el uso de mascarillas ^10^.

### Respuesta de los servicios de salud

Todos los países presentaron una brecha entre la disponibilidad de camas UCI y ventiladores, y la demanda generada por la pandemia. Perú fue el país con la menor disponibilidad de estos recursos (tabla 1) ^9^. Sin embargo, estuvo entre los que mayor incremento lograron: 349% en camas UCI y 83% en ventiladores en los primeros 100 días de la pandemia ^9^. El suministro de oxígeno fue otro problema crítico. Brasil llegó a necesitar 340 000 cilindros de oxígeno por día; Argentina, Colombia, México y Perú requirieron aproximadamente 100 000 cilindros cada uno, siendo frecuente que las familias asumieran los costos ^11^.

**Tabla 1:** Recursos de los sistemas de salud de cinco países seleccionados al inicio de la pandemia.

Indicador	Brasil	Chile	Colombia	Ecuador	Perú
Médicos x 100 000 hab.	1,8	2,5	2,2	2,0	1,3
Enfermeros x 1 000 hab.	1,5	2,7	1,3	2,5	2,4
Camas x 1 000 hab.	2,3	2,1	1,7	1,5	1,6
Camas UCI x 100 000 hab.	17,0	5,2	10,8	6,8	2,5
Ventiladores x 100 000 hab.	29,6	6,8	10,8	10,5	0,9

Adaptado de Benítez *et al.* ^(^^9^^)^.

UCI: Unidad de cuidados intensivos.

Con respecto a las pruebas de diagnóstico, Chile y Perú registraron el mayor número de pruebas diarias por población entre abril y mayo de 2020. Ambos países contaban inicialmente con un solo laboratorio de biología molecular; sin embargo, para agosto de 2020, el número de laboratorios habilitados se incrementó a 112 en Chile y 25 en Perú ^9^.

Al inicio de la pandemia, se generalizó el uso de medicamentos con supuesto efecto frente a la COVID-19, como ivermectina, cloroquina y azitromicina, basados en escasa evidencia. Pese a que más adelante se contó con evidencia sólida en contra de su uso, esta práctica se mantuvo de forma extendida ^11^. En Perú, el 52,2% de las personas con síntomas de COVID-19 no acudió a ningún servicio de salud durante el primer año de la pandemia. Entre los principales factores asociados a esta conducta se identificaron la residencia en zonas de selva urbana o rural, mientras que factores como tener más de 50 años, contar con educación secundaria o superior y disponer de seguro de salud se comportaron como protectores ^12^.

Respecto al gasto de bolsillo y el gasto catastrófico en salud, un estudio comparativo entre México y Perú evidenció que la proporción de hogares con gasto catastrófico aumentó en ambos países durante la pandemia (México: 3,3% a 5,6%; Perú: 8,7% a 9,6%), con niveles consistentemente más altos en Perú ^13^^,^^14^. En este último, los principales factores asociados a un mayor gasto de bolsillo fueron el tipo de aseguramiento (particularmente el Seguro Integral de Salud), la presencia de enfermedades en el hogar, un resultado positivo para COVID-19 y la presencia de adultos mayores en el hogar ^14^.

Los elementos que mejor funcionaron para mejorar la respuesta del sistema de salud en la región incluyeron: entrenamiento de recursos humanos, coordinación público-privada, sistemas robustos de vigilancia epidemiológica, rápida construcción de hospitales de campo, reconversión de camas hospitalarias, escalamiento de capacidades de laboratorio y mecanismos de coordinación nacional con base científica ^15^.

### Telesalud

La pandemia provocó una reducción significativa del uso de los servicios de salud, especialmente del primer nivel de atención, a consecuencia de la cuarentena y el cierre de establecimientos. Para compensar este déficit, los países impulsaron o fortalecieron sus servicios de telemedicina; en este contexto, Perú y Chile destacaron por el mayor desarrollo de esta estrategia ^9^. Las tecnologías empleadas fueron diversas, incluyendo llamadas telefónicas, videoconferencias, aplicaciones móviles, correos electrónicos, mensajes de texto, páginas web y software de teleradiología. Estas herramientas se usaron con fines de prevención, tamizaje, triaje, diagnóstico, tratamiento y seguimiento. No obstante, su implementación enfrentó múltiples barreras, principalmente relacionadas con la conectividad, la alfabetización digital y los marcos regulatorios ^16^.

### Respuesta de apoyo social

La pandemia se desarrolló en un contexto de alta desigualdad social y económica. América Latina es una de las regiones más inequitativas del mundo, donde coexisten grupos altamente vulnerables: migrantes no documentados, población rural e indígena, afrodescendientes y hogares de bajos ingresos. Un grupo de expertos recomendó mejorar la protección social y económica para estos grupos de manera focalizada y culturalmente pertinente ^15^. En particular, entre los migrantes venezolanos se registraron algunas de las coberturas de vacunación más bajas de la región, lo que evidencia brechas persistentes en el acceso a los servicios de salud.

### Infodemia

La circulación masiva de información falsa y no verificada generó incertidumbre en la población sobre las medidas de prevención, el manejo terapéutico y la real situación de la pandemia. La mayoría de los países implementó estrategias para combatir la infodemia, aunque con alcances diferentes. Argentina desarrolló la plataforma web “Confiar”, que incluía secciones para verificar afirmaciones, identificar noticias falsas en circulación y orientar sobre cómo detectar desinformación. Por su parte, Brasil, habilitó un número de WhatsApp para consultas respondidas por equipos técnicos ^17^.

En contraste, Perú fue uno de los pocos países que no adoptó medidas explícitas para abordar la infodemia, limitándose a anunciar sanciones penales de dos a cuatro años de prisión por la difusión de noticias falsas relacionadas con la pandemia ^18^. A pesar de ello, una evaluación del índice de riesgo de infodemia posicionó a Perú con el nivel de riesgo más alto de la región (99,1%) ^10^. Un análisis de las causas y contramedidas para enfrentar este problema propone un marco amplio que incluye la alfabetización en salud, el trabajo coordinado con plataformas de medios masivos, la comunicación de riesgo mediante voceros acreditados y la orientación clara a la población ^19^.

### Vacunación

Las vacunas contra la COVID-19 tardaron en llegar a Latinoamérica y el Caribe. Durante el primer semestre de 2021, las coberturas de vacunación en la región fueron significativamente menores que en Europa y Norteamérica, principalmente por la limitada disponibilidad de dosis. No obstante, en etapas posteriores, la región alcanzó una de las mayores coberturas de vacunación completa (dos dosis) a nivel mundial ^11^. En Perú, con el objetivo de ampliar el acceso a la vacuna, el Proyecto LEGADO implementó centros de vacunación en diversos puntos, logrando niveles de satisfacción superiores al 90% entre los usuarios ^20^.

### Innovación e investigación

En Perú, el Consejo Nacional de Ciencia, Tecnología e Innovación (CONCYTEC) convocó a fondos concursables de investigación orientados al control de la pandemia, alcanzando una inversión de USD 2,9 millones. Los principales tópicos financiados incluyeron innovación tecnológica, telemedicina y salud móvil, así como estudios epidemiológicos y sociales. Sin embargo, no se ha evaluado formalmente el impacto de estos proyectos en el control de la pandemia, ni en la preparación ante futuras emergencias sanitarias ^21^.

Una de las innovaciones más destacadas fue el diseño del concentrador de oxígeno COVOX, desarrollado conjuntamente por la Pontificia Universidad Católica del Perú (PUCP) en colaboración con la empresa DIACSA. Este dispositivo libera oxígeno a una tasa de flujo de 15 L/min (±1,5), superior a los concentradores convencionales de alto flujo (10 L/min), lo que contribuyó a mitigar la escasez de oxígeno durante los momentos críticos de la pandemia ^22^. En contraste, países como India gestionaron fondos estratégicos para investigación e innovación, promovieron alianzas gobierno-academia-industria y lograron aportes significativos en kits de diagnóstico, vacunas y medicamentos. Además, sus investigadores generaron alrededor del 8% de las revisiones sistemáticas y metaanálisis a nivel global durante el primer año de la pandemia ^23^.

### Mortalidad por COVID-19

Según datos de la OMS (4), Perú fue el país sudamericano con mayor exceso de mortalidad por cada 100 000 habitantes durante 2020 y 2021, seguido por Bolivia y Ecuador (figura 1). Esta situación estuvo directamente relacionada con la menor disponibilidad inicial de recursos críticos (especialmente ventiladores mecánicos y camas UCI) en comparación con otros países de la región.

**Figura 1:** Exceso de mortalidad por 100 000 habitantes, 2020-2021 en países seleccionados de Sudamérica.

Fuente: Organización Mundial de la Salud. Global excess deaths associated with COVID-19 (5).

## DISCUSIÓN

Las respuestas de los países sudamericanos a la pandemia de COVID-19 fueron variadas en oportunidad, alcance e impacto, con resultados dispares en mortalidad que reflejan diferencias estructurales previas en sus sistemas de salud. Un factor determinante fue el nivel de preparación del sistema de salud antes del inicio de la pandemia. Perú, que presentaba la menor tasa de ventiladores mecánicos por habitante de la región, registró la mayor mortalidad; en contraste, países mejor equipados con recursos críticos mostraron mejores resultados. Se ha identificado que los principales recursos que definen la preparación frente a pandemias se incluyen la disponibilidad de camas hospitalarias y de cuidados intensivos, ventilación mecánica, recursos humanos en salud, medicamentos, vacunas, capacidad diagnóstica y seguimiento de contactos ^21^.

Las intervenciones no farmacológicas, en particular el distanciamiento social, el confinamiento y el uso de mascarillas, fueron las medidas con mayor consistencia en el control de la transmisión según la evidencia disponible ^22^. No obstante, su efectividad estuvo limitada por factores socioeconómicos, como la alta informalidad laboral en Perú, que comprometió el cumplimiento del confinamiento.

La pandemia también evidenció el potencial de la telesalud como componente estratégico de la respuesta sanitaria, especialmente en la atención de enfermedades crónicas y el seguimiento de casos. El uso de estas tecnologías durante la pandemia fue amplio y variado, y existen múltiples barreras por superar para su extensión sostenida, incluyendo conectividad, regulación y alfabetización digital ^24^.

La infodemia emergió como un fenómeno paralelo a la pandemia, con consecuencias directas en el comportamiento de la población y el uso de medicamentos sin evidencia. El caso peruano es paradigmático: siendo el país con mayor riesgo de infodemia de la región, careció de estrategias explícitas para combatirla. Esto contrasta con la experiencia de Argentina y Brasil, que desarrollaron herramientas activas de comunicación verificada ^16^. En este contexto, la gestión de la información en salud pública debe integrarse como un componente esencial en los planes de respuesta a pandemias.

En cuanto a innovación e investigación, se observaron diferencias sustantivas entre países. Mientras que India integró la investigación como pilar de su respuesta, los países de la región, incluido Perú, tuvieron aportes limitados. La experiencia del concentrador COVOX demuestra el potencial de la innovación local, pero su escalamiento requirió mayor articulación entre academia, industria y gobierno ^20^^,^^23^.

Finalmente, el liderazgo y la gobernanza fueron factores clave en la conducción de la respuesta. Países asiáticos, con experiencia previa en epidemias, como el SARS de 2003, mostraron mayor efectividad, particularmente aquellos con administración pública eficiente y con comités técnicos de gestión de pandemia ^25^. La complejidad de la crisis sanitaria hace pertinente replantear los modelos de liderazgo vertical hacia esquemas más horizontales, interdisciplinarios e intersectoriales.

## LECCIONES APRENDIDAS

La experiencia de la pandemia de COVID-19 en Sudamérica aporta un conjunto de enseñanzas que deben orientar la preparación para futuros eventos sanitarios de gran magnitud:

1. La infraestructura crítica de salud (camas UCI, ventiladores, suministro de oxígeno y laboratorios de diagnóstico) constituye una condición necesaria para responder eficazmente a una pandemia. La brecha estructural preexistente en Perú fue un factor determinante de su elevada mortalidad. La inversión en esta infraestructura no debe ser reactiva sino sostenida.
2. Las intervenciones no farmacológicas, especialmente el confinamiento y el distanciamiento social, son efectivas para controlar la transmisión, pero su efectividad está condicionada por factores socioeconómicos como la informalidad laboral. Las políticas sanitarias deben articularse con políticas económicas y sociales de protección.
3. La telesalud demostró ser una herramienta valiosa para garantizar la continuidad de la atención durante la pandemia. Su institucionalización como componente permanente del sistema de salud (y no como respuesta de emergencia) requiere marcos regulatorios, inversión en conectividad y formación del personal.
4. La infodemia es un fenómeno tan peligroso como la propia epidemia. La ausencia de estrategias activas de comunicación de riesgos y gestión de la desinformación en Perú contribuyó al uso masivo de medicamentos sin evidencia y al incumplimiento de medidas preventivas. La comunicación en salud pública debe integrarse como eje transversal de la respuesta.
5. La vacunación, pese a los retrasos en el acceso a dosis, fue la intervención de mayor impacto en la reducción de la mortalidad. La equidad en el acceso a vacunas (incluyendo a poblaciones vulnerables como migrantes y comunidades indígenas) debe incorporarse explícitamente en los planes de inmunización.
6. La investigación e innovación local, cuando están articuladas con las necesidades de la respuesta, pueden generar soluciones de alto impacto. Sin embargo, estas iniciativas requieren planificación, financiamiento sostenido y mecanismos de traducción de la evidencia en política pública.
7. El liderazgo técnico y político, la coordinación intersectorial y la gobernanza basada en evidencia fueron determinantes en los países con mejor desempeño. La pandemia evidenció la necesidad de instalar y fortalecer comités técnicos permanentes de gestión de riesgos sanitarios.

## RECOMENDACIONES

A partir de las lecciones aprendidas, se formulan las siguientes recomendaciones para fortalecer la preparación del Perú y los países de la región frente a futuras pandemias:

1. Cerrar la brecha de infraestructura crítica de salud. Perú debe establecer metas concretas y financiadas para incrementar sus ratios de camas UCI, ventiladores mecánicos y capacidad de laboratorio, tomando como referencia los estándares de la OCDE y la experiencia comparada regional.
2. Institucionalizar la telesalud como componente permanente del sistema. Esto implica desarrollar marcos regulatorios actualizados, garantizar la interoperabilidad de sistemas de información, invertir en infraestructura de conectividad en zonas rurales y capacitar al personal de salud.
3. Desarrollar una estrategia nacional de comunicación de riesgos e infodemia. El país debe contar con una plataforma oficial de verificación de información, vocerías técnicas acreditadas y programas de alfabetización en salud para la población general y grupos vulnerables.
4. Fortalecer la protección social para grupos vulnerables. Los planes de respuesta a pandemias deben incluir componentes específicos de protección económica y social para trabajadores informales, migrantes, comunidades indígenas y adultos mayores.
5. Crear un fondo permanente de investigación e innovación en salud pública. El Perú debe contar con financiamiento estable para la investigación orientada a la preparación y respuesta ante emergencias sanitarias, promoviendo alianzas entre gobierno, academia e industria.
6. Establecer un Comité Técnico Nacional Permanente de Preparación ante Epidemias y Pandemias, con mandato técnico claro, independencia del ciclo político y capacidad de activación inmediata en contextos de emergencia. Este comité debería actualizar periódicamente el Plan Nacional de Respuesta a Pandemias.
7. Promover la equidad como principio rector de la respuesta sanitaria. Las intervenciones de salud pública (diagnóstico, tratamiento, vacunación, apoyo social) deben diseñarse con enfoque de equidad, garantizando el acceso de todas las poblaciones, especialmente las más vulnerables.

## CONCLUSIONES

La pandemia de COVID-19 evidenció las brechas estructurales de los sistemas de salud de Sudamérica, siendo Perú el país más afectado en términos de mortalidad. La heterogeneidad de respuestas entre países permitió identificar factores protectores y prácticas replicables como mayor disponibilidad de recursos críticos, liderazgo técnico articulado con evidencia, intervenciones no farmacológicas oportunas, estrategias activas para combatir la infodemia, adopción acelerada de la telesalud e inversión en investigación e innovación local.

Cinco años después del inicio de la pandemia, el Perú enfrenta el imperativo de transformar las lecciones aprendidas en políticas públicas concretas. La preparación frente a futuras pandemias no puede basarse únicamente en respuestas reactivas; requiere inversión sostenida en infraestructura, recursos humanos, sistemas de vigilancia, comunicación de riesgos y protección social. El escenario de una nueva pandemia de influenza aviar u otro agente emergente no es improbable, y el costo de la inacción ha sido ya demostrado en vidas humanas.

La pandemia también demostró que, aunque es posible reducir la transmisión y desarrollar vacunas con rapidez sin precedentes a nivel mundial, esto no evita que la demanda de servicios críticos supere la capacidad de respuesta de sistemas de salud débiles. Por ello, el fortalecimiento del sistema de salud peruano debe ser una prioridad de Estado, construida sobre la experiencia adquirida, la evidencia científica y el compromiso con la equidad en salud.
